# Initial Evaluation of Therapy Response after Adjuvant Radioiodine Therapy in Patients with Early-Stage Papillary Thyroid Cancer—Does Time Matter?

**DOI:** 10.3390/cancers14030501

**Published:** 2022-01-20

**Authors:** Freba Grawe, Yuri Cramm, Matthias P. Fabritius, Johannes Ruebenthaler, Viktoria F. Koehler, Harun Ilhan, Peter Bartenstein, Vera Wenter, Andrei Todica

**Affiliations:** 1 Department of Nuclear Medicine, University Hospital, LMU Munich, 81377 Munich, Germany; Freba.Ahmaddy@med.uni-muenchen.de (F.G.); yuri.cramm@gmail.com (Y.C.); Harun.Ilhan@med.uni-muenchen.de (H.I.); Peter.Bartenstein@med.uni-muenchen.de (P.B.); Vera.Wenter@med.uni-muenchen.de (V.W.); 2 Department of Radiology, University Hospital, LMU Munich, 81377 Munich, Germany; Matthias.Fabritius@med.uni-muenchen.de (M.P.F.); Johannes.Ruebenthaler@med.uni-muenchen.de (J.R.); 3 Department of Internal Medicine IV, University Hospital, LMU Munich, 81377 Munich, Germany; Viktoria.Koehler@med.uni-muenchen.de; 4 Department of Medicine I, Goethe University Hospital, 60596 Frankfurt, Germany; 5 Die Radiologie, 80331 Munich, Germany

**Keywords:** PTC, diagnostic control, radioiodine therapy, rhTSH

## Abstract

**Simple Summary:**

In recent years, there has been a clear trend toward personalized therapy procedures in patients with thyroid cancer with the aim to avoid unnecessary overtreatment of patients and to ensure an improved quality of life. We confirmed that early diagnostic control at 6 months after initial radioiodine therapy shows no significant disadvantages compared to a delayed control after 9 months. Further, it was observed that patients stimulated by hormone withdrawal before radioiodine therapy had significantly better outcomes compared to patients stimulated exogenously with recombinant human thyroid-stimulating hormone (rhTSH). However, early diagnostic control after TSH stimulation represents the most balanced solution for the patient, specifically regarding hypothyroidism symptoms after hormone withdrawal.

**Abstract:**

Background: The aim was to assess ablation success after initial radioiodine (RAI) therapy in early-stage PTC patients and compare outcomes of first diagnostic control after 6 and 9 months (6m/9m-DC) to examine whether time could possibly avoid unnecessary overtreatment. Methods: There were 353 patients who were matched regarding age, sex, and tumor stage and divided in two groups depending on time of first DC (6m- and 9m-DC). Therapy response was defined as thyroglobulin level <0.5 ng/mL, no pathological uptake in the diagnostic I-131 whole-body scintigraphy (WBS), and no further RAI therapy courses. The 6m-DC group was further divided into endogenously and exogenously stimulated TSH before RAI therapy and compared regarding outcome. Results: No significant differences were found between 6m-DC vs. 9m-DC regarding I-131 uptake in WBS (*p* = n.s.), Tg levels (*p* = n.s.), re-therapy rates (*p* = n.s.), and responder rates (*p* = n.s.). Significantly less relevant pathological I-131 uptake was found in WBS (*p* = 0.006) in endogenously compared to exogenously stimulated 6m-DC patients, resulting in lower re-therapy (*p* = 0.028) and higher responder rates (*p* = 0.001). Conclusion: DC at 6 months after RAI therapy and stimulation with recombinant human thyroid-stimulating hormone (rhTSH) represent the most balanced solution. Particularly regarding quality of life and mental relief of patients, early DC with rhTSH represents sufficient and convenient assessment of ablation success.

## 1. Introduction

Differentiated thyroid carcinoma (DTC) is the most common malignant endocrine neoplasm with increasing incidence worldwide [1]. Papillary thyroid carcinoma (PTC) presents the most common histological subtype and shows a 5-year survival rate of over 95% [2]. The established therapeutic approach includes a multimodal therapy with initial (hemi-)thyroidectomy and, depending on tumor stage, an additional radioiodine (RAI) therapy to eliminate residual benign thyroid tissue and undetectable microscopic tumor tissue [3]. In recent years, there has been a clear trend toward personalized therapy procedures with the aim to avoid unnecessary overtreatment of patients and to ensure an improved quality of life. However, an adequate aftercare is crucial in these patients, since tumor recurrence can occur after many years [4].

To evaluate ablation success, a diagnostic control, including a I-131 whole-body scintigraphy (WBS) under TSH stimulation, neck ultrasound, and laboratory examination, is recommended, which should be performed 6 to 12 months after RAI therapy (first diagnostic control (DC)), depending on the respective guidelines. The optimal time to perform DC to evaluate ablation success after initial radioiodine therapy in DTC patients is widely discussed among experts and results in different recommendations in the corresponding German, British, and American guidelines [3,5,6]. One advantage of an early first diagnostic control (i.e., after 6 months) would be an earlier emotional and mental relief due to first completion of therapy resulting in an overall improved quality of life, an aspect in tumor patients that should not be underestimated. A further decisive reason is that pregnancy should be strictly avoided for a certain period after exposure to radioactive iodine. Extending the period between RAI therapy and first diagnostic control also delays a desired pregnancy. This factor may play an important role in women of childbearing age, especially given the 3:1 ratio of women to men with DTC [7]. From therapy of benign thyroid diseases, in which lower activity doses are applied than in thyroid carcinoma, it is known that the effect of radioiodine in the treatment of hyperthyroidism can last well over a year [8]. Therefore, we hypothesized that if diagnostic control is performed too early in DTC patients, there may be a risk that the full effect of initial RAI therapy is not achieved and possible remaining thyroid tissue is not completely ablated, resulting in a possibly unnecessary re-therapy of patients.

The aim of our study was to assess ablation success after initial RAI therapy in early-stage PTC patients receiving a “low-dose” radioiodine therapy of approx. 2000 MBq (54 mCi) I-131 and to compare the outcome of first diagnostic control after 6 months and after 9 months in order to examine whether time could possibly avoid unnecessary overtreatment of patients.

## 2. Materials and Methods

### 2.1. Study Patients

We retrospectively reviewed 1693 consecutive patients with DTC from our institutional database. Only patients with early stage PTC (pT1a (m, multifocal)/pT1b/pT2, N0, R0, M0) were included in this study, who underwent total thyroidectomy followed by RAI therapy according to German guidelines [9] in the Department of Nuclear Medicine (University Hospital, LMU Munich) between May 2013 and December 2018. Epidemiological and clinical features of these patients were assessed (e.g., age at diagnosis, gender, TNM stage, tumor size), and patients were matched regarding primary presentation (TNM stage, age, sex). TNM stage was classified according to the seventh edition of the American Joint Committee on Cancer [10]. All patients underwent total thyroidectomy with or without lymphadenectomy, followed by (adjuvant) initial RAI therapy. Prior to RAI therapy, patients were stimulated with recombinant human thyroid-stimulating hormone (rhTSH, Thyrogen^®^, Sanofi Genzyme, Cambridge, MA, USA) i.m. on 2 consecutive days or underwent hormone withdrawal prior to RAI therapy to achieve TSH levels ≥30 µU/mL according to current guideline recommendations [3]. According to the institutional risk-adapted dose scheme, all early-stage PTC patients were treated with a mean of 2000 MBq (54 mCi) I-131 at initial RAI therapy.

### 2.2. Follow-Up Examinations and First Diagnostic Control

Follow-up examinations, including a cervical ultrasound and laboratory examination in patients with inconspicuous follow-up, were usually performed every 3 months in the first year, every 6 months in the second year, and annually thereafter. At first diagnostic control (DC) at 6 or 9 months after initial RAI therapy, additionally to laboratory measurements, a diagnostic I-131 whole-body scintigraphy (WBS) was performed approximately 72 h after application of approx. 370 MBq I-131 (10 mCi) in hypothyroidism (TSH levels ≥ 30 µU/mL) or after administration of rhTSH i.m. on 2 consecutive days. I-131 uptake in the WBS was assessed retrospectively by experts; four experienced nuclear medicine physicians re-evaluated I-131 uptake in the WBS without knowledge of the medical reports. Tg and Tg recovery were measured under stimulation, respectively, 3 days after the last rhTSH injection. Neck ultrasonography was performed in all patients but not further evaluated in this study. Patients were classified as responders to adjuvant RAI therapy if stimulated Tg levels were lower than 0.5 ng/mL and non-pathological cervical or distant I-131 uptake was seen in the WBS. In contrast, if Tg was >0.5 ng/mL or pathological uptake was detected in the WBS leading to an additional RAI therapy, the first adjuvant RAI therapy was considered as inadequate.

### 2.3. Group and Subgroup Analysis

To compare outcome of ablation success in PTC patients at different time points, patients were firstly divided into two groups depending on the time of first DC. Patients of an historical, institutional collective receiving first DC at 6 months after initial RAI therapy (6m-DC) were compared to a newer patient cohort receiving first DC at 9 months after initial RAI therapy (9m-DC). Patients were matched regarding age, sex, and tumor stage. Due to non-homogenously distributed patients regarding TSH stimulation at initial RAI therapy in the 6m-DC group, a subgroup analysis was performed comparing endogenous vs. exogenous TSH stimulation (6m-DC-endo vs. 6m-DC-exo). Since the newer patient cohort was exclusively stimulated by rhTSH for RAI therapy, the 9m-DC (9m-DC-exo) group consisted only of patients receiving the initial RAI therapy under exogenous stimulation, whereas the 6m-DC group consisted of both, endogenously (6m-DC-endo) and exogenously stimulated patients (6m-DC-exo).

### 2.4. Statistical Analysis

All continuous variables were expressed as mean standard deviation (SD). The Mann–Whitney U test was used to compare metric variables. A Chi-squared test was used to compare categorial variables. All analyses were performed using SPSS computer software (SPSS Statistics 25, IBM, Armonk, NY, USA).

## 3. Results

### 3.1. Study Patients

In this study, 353 patients (276 female, 78%) were included. The mean age at diagnosis was 48.5 ± 13.7 years. Most patients had pT1a(m)-stage or pT1b-stage (279/353, 79%), followed by pT2-stage (74/353, 21%). The histological subtype was classical PTC in 69% of patients (*n* = 245/353) and the follicular variant of PTC in 31% (*n* = 108/353). Multifocality was observed in 46% of patients (*n* = 161/353), whereas in 52% of patients only one lesion was detected (*n* = 185/353). In seven patients no information on multifocality was found. Mean tumor size was 13.81 ± 8.29 mm. Data on tumor size were missing for 12 patients.

The mean-administered I-131 dose activity at initial RAI therapy was 2042.5 ± 45.0 MBq (55.2 ± 1.2 mCi). Most of the patients were stimulated with rhTSH (263/353, 75%).

### 3.2. Group Analysis

In 204/353 (58%) patients, DC was evaluated at 6 months after initial RAI therapy (6m-DC) and in 149/353 (42%) patients at 9 months after initial RAI therapy (9m-DC). Both groups were matched regarding age (49.3 ± 13.3 years in 6m-DC vs. 47.3 ± 14.2 years in 9m-DC, *p* = 0.181), sex (82% in 6m-DC (167/204) vs. 73% in 9m-DC (109/149), *p* = 0.050), and tumor stage (T1a(m)/T1b-stage: 80% in 6m-DC (163/204) vs. 78% in 9m-DC (116/149), *p* = 0.640). All 9m-DC patients were stimulated with rhTSH at initial RAI therapy, whereas only around half of the 6m-DC patients received rhTSH at initial RAI therapy (56% in 6m-DC (114/204) vs. 100% in 9m-DC (149/149), *p* = 0.001). Stimulated Tg level before RAI therapy was comparable in both groups (2.7 ± 3.9 ng/mL in 6m-DC vs. 4.1 ± 10.9 ng/mL in 9m-DC, *p* = 0.105). Patient characteristics of the group analysis are demonstrated in Table 1.

At initial DC, no significant differences were found among groups regarding Tg responder rates after TSH stimulation (90% in 6m-DC (184/204) vs. 88% in 9m-DC (131/149), *p* = 0.495). Furthermore, non-pathological cervical or distant I-131 uptake in the WBS based on expert opinion was somewhat lower in the 9m-DC group but without reaching statistical significance (84% in 6m-DC (172/204) vs. 78% in 9m-DC (116/149), *p* = 0.112). Overall, in the clinical routine, an additional RAI therapy cycle was not performed nearly in the same proportion of patients in both groups, albeit with a similar trend to a worse outcome in the 9m-DC group (86% in 6m-DC (176/204) vs. 79% in 9m-DC (118/149), *p* = 0.078) resulting in similar outcome of the overall responder rates (75% in 6m-DC (152/204) vs. 67% in 9m-DC (100/149), *p* = 0.129).

### 3.3. Subgroup Analysis

In the subgroup analysis, 90/204 (44%) patients were included in the 6m-DC-endo and 114/204 (56%) patients in the 6m-DC-exo group. Subgroups were matched regarding age (49.2 ± 12.0 years in 6m-DC-endo vs. 49.4 ± 14.4 years in 6m-DC-exo, *p* = 0.920), sex (81% in 6m-DC-endo (73/90) vs. 83% in 6m-DC-exo (94/114), *p* = 0.804), and tumor stage (pT1a(m)/pT1b-stage: 79% in 6m-DC-endo (71/90) vs. 81% in 6m-DC-exo (92/114), *p* = 0.748). Stimulated Tg level before RAI therapy was comparable in both groups (3.2 ± 3.9 ng/mL in 6m-DC-endo vs. 2.4 ± 3.9 ng/mL in 6m-DC-exo, *p* = 0.150). Patient characteristics of the subgroup analysis are demonstrated in Table 2.

At initial DC, Tg responder rates after TSH stimulation were comparable in both groups (94% in 6m-DC-endo (85/90) vs. 87% in 6m-DC-exo (99/114), *p* = 0.070). Non-pathological I-131 uptake in the WBS was significantly lower in the 6m-DC-endo group compared to the 6m-DC-exo (92% in 6m-DC-endo (83/90) vs. 78% in 6m-DC-exo (89/114), *p* = 0.006). The same was observed for re-therapy rates: 6m-DC-endo patients needed, significantly less often, further RAI therapy cycles (92% in 6m-DC-endo (83/90) vs. 82% in 6m-DC-exo (93/114), *p* = 0.028). The overall responder rates at the first DC were consequently significantly higher in the 6m-DC-endo group compared to the exogenously stimulated group (87% in 6m-DC-endo (78/90) vs. 65% in 6m-DC-exo (74/114), *p* = 0.001).

## 4. Discussion

To our knowledge, this is the first study comparing ablation success (DC) after initial RAI therapy at 6 months versus 9 months in early-stage PTC patients. Due to the lower activity doses applied in early-stage PTC patients, we hypothesized that early DC can lead to not fully ablated tissue in the short period of time, resulting in higher non-responder rates after initial RAI therapy, and should be, therefore, delayed. However, our results showed no significant differences at first DC between both groups (6m-DC vs. 9m-DC) regarding I-131 uptake in the WBS, Tg levels, re-therapy rates, and overall responder rates. This finding is crucial in clinical routine since the possibility to perform after-care examinations at an earlier point in time enables patients to gain emotional and mental relief earlier. Furthermore, for women of childbearing age early, completion of therapy may have significant impact due to strict contraception for a certain period after RAI therapy.

Due to non-homogenously distributed patients regarding TSH stimulation at initial RAI therapy in the 6m-DC group, we performed a subgroup analysis comparing endogenously vs. exogenously stimulated TSH before initial RAI therapy. We found significantly fewer patients with a relevant I-131 uptake in the WBS in the group undergoing thyroid hormone withdrawal (6m-DC-endo) compared to patients with exogenously stimulated TSH (6m-DC-exo) before RAI therapy, resulting in significantly lower re-therapy and consequently higher responder rates in these patients. As the effectiveness of RAI therapy is presumably dependent on the TSH level, TSH stimulation increases the I-131 uptake and, therefore, plays a major role in RAI therapy. The use of rhTSH in stimulating the uptake of I-131 in non-metastasized DTC patients was already established and approved by the European Medicines Agency (http://www.ema.europa.eu, accessed on 4 January 2022) in 2005 and the United Stated Food and Drug Administration (http://www.fda.gov, accessed on 4 January 2022) in 2007; its comparability to hormone withdrawal was already shown in previous studies [11,12,13]. Our findings in favor of hormone withdrawal might be explained by a somewhat reduced renal function in hypothyroidism, which leads to lower clearance rates and higher half-life of I-131 in the blood, resulting in a proportional increase of I-131 activity dose in remnant tissue [14,15]. Further, the ratio of a therapeutically usable I-131 activity dose in remnant tissue compared to the radiation exposure of the rest of the body appears to be more favorable under endogenous stimulation than under rhTSH stimulation.

The HiLo trial by Mallick et al. demonstrated comparable effectiveness of hormone withdrawal and rhTSH in patients treated with low-dose (1.1 GBq) vs. high-dose (3.7 GBq) RAI therapy, though with higher success rates compared to our results in both the rhTSH stimulated group (87%) and the hormone withdrawal group (87%) [16]. This finding can be explained by the different study designs: A negative scan was defined as an uptake of <0.1%, whereas our scan was classified visually as negative/positive. Furthermore, the Tg cutoff was 2.0 ng/mL in the HiLo study, which was 0.5 ng/mL in our study, leading to an overall more stringent study design. For further analysis, the cervical I-131 uptake was measured additionally manually in the WBS, where results showed lower mean I-131 uptake in the 6m-DC-endo group compared to the 6m-DC-exo and 9m-DC group but without reaching statistical significance. The partially lower ablation success rates in our study (87% in 6m-DC-endo vs. 65% in 6m-DC-exo) compared the HiLo study (87.1% in rhTSH vs. 86.7% in hormone withdrawal group) can be, therefore, explained by the higher rates of relevant I-131 uptake resulting in higher re-therapy rates.

A study by Carvalho et al. reported on only a marginal risk of recurrence in 1420 DTC patients if patients showed negative WBS and Tg levels at 12 months after RAI therapy [17].

However, thyroid hormone withdrawal is associated with many adverse effects and can significantly affect quality of life [18,19]. Possible hypothyroidism symptoms including cognitive impairment, emotional dysfunction, and physical discomfort can be avoided with the use of rhTSH, which has important advantages [19]. Scheduling of patients and shorter hospital stay are in favor of rhTSH, especially concerning the higher rates of women to men. Especially women with children benefit from the ability to function physically and socially at home and work [20]. Shroeder et al. examined the impact of short-term hypothyroidism on the health-related quality of life of patients after rhTSH vs. hormone withdrawal and reported on a significant decline in quality of life after hormone withdrawal and emphasized the facilitated diagnostic testing by rhTSH stimulation [21]. In the HiLo trial, the combination of exogenously stimulated TSH and low-dose RAI therapy showed best quality of life and should be preferred [16]. Furthermore, radiation exposure is lower after rhTSH due to the faster clearance, as mentioned above [22].

Regarding Tg responder rates, in the subgroup analysis comparing 6m-DC-endo with 6m-DC-exo, our results showed comparable rates, though a trend toward higher Tg responder rates in the endogenously stimulated group was observed (6m-DC-endo 94% vs. 6m-DC-exo 87%). However, long-term data with an observation period of 10 years demonstrated equal survival time regardless of TSH stimulation by rhTSH or thyroid hormone withdrawal.

In this study, only patients with early-stage PTC (multifocal T1N0M0 and T2N0M0) were included, and therapy was performed according to German guidelines including total thyroidectomy and RAI therapy [23]. It must be noted that, in the widely used ATA (American thyroid association) guidelines and according to the European consensus for the management of patients with DTC, patients are treated less aggressively and RAI therapy after thyroidectomy is not routinely recommended [5,24].

There are some limitations to this study. Firstly, there may have been a selection bias because of the retrospective design. Secondly, patients of an historical, institutional collective were compared to a newer patient cohort; therefore, a corresponding endogenously stimulated patient cohort with DC at 6 months after RAI therapy is consequently missing. Furthermore, it should be emphasized that results of this early-stage patient cohort cannot be transferred to patients with high-risk PTC.

Thyroglobulin antibodies are only present in a minority of the patients and, therefore, could not be included in the analysis in a convincing way. This is indeed a limitation of the study, which cannot be overcome. However, although the recovery is less sensitive than the direct measurement of the antibodies, we are convinced that an undisturbed recovery adds confidence for the validity of the measured thyroglobulin. The diagnostic impact of cervical ultrasound was not assessed.

## 5. Conclusions

Our overall results led to the conclusion that early DC at 6 months after RAI therapy with rhTSH represents the most balanced solution considering possible additional information provided by DC after withdrawal of thyroid hormone weighed against symptoms of hypothyroidism. Particularly with regard to quality of life and mental relief of patients, early DC after stimulation with rhTSH represents a sufficient and convenient assessment of ablation success.

## Figures and Tables

**Table 1:** Patient characteristics of groups (6m-DC vs. 9m-DC).

*n* = 353	6m-DC, *n* = 204	9m-DC, *n* = 149	*p*-Value
Age (yrs)	49.3 ± 13.3	47.3 ± 14.2	0.181
Female—no. (%)	167/204 (82%)	109/149 (73%)	0.050
pT stage—no. (%)			0.640
pT1a(m)/pT1b	163/204 (80%)	116/149 (78%)	
pT2	41/204 (20%)	33/149 (22%)	
TSH stimulation with rhTSH—no. (%)	114/204 (56%)	149/149 (100%)	0.001
Tg level (ng/mL)	2.7 ± 3.9	4.1 ± 10.9	0.105

M, months; DC, diagnostic control; p, pathological; T, tumor; (m), multifocal; TSH, thyroid-stimulating hormone; Tg, thyroglobulin.

**Table 2:** Patient characteristics of subgroups (6m-DC-endo vs. 6m-DC-exo).

*n* = 214	6m-DC-endo, *n* = 90	6m-DC-exo, *n* = 114	*p*-Value
Age (yrs)	49.2 ± 12.0	49.4 ± 14.4	0.920
Female—no. (%)	73/90 (81%)	94/114 (83%)	0.804
pT stage—no. (%)			0.748
pT1a(m)/pT1b	71/90 (79%)	92/114 (81%)	
pT2	19/90 (21%)	22/114 (19%)	
Tg level (ng/mL)	3.2 ± 3.9	2.4 ± 3.9	0.150

M, months; DC, diagnostic control; endo, endogenous; exo, exogenous; p, pathological; T, tumor; (m), multifocal; TSH, thyroid-stimulating hormone; Tg, thyroglobulin.

## Data Availability

The data presented in this study are available upon reasonable request from the corresponding author.
